# Age Differences in Awe Among Young, Middle-aged, and Older Adults: Extensions of Socioemotional Selectivity Theory

**DOI:** 10.1093/geroni/igab046.2839

**Published:** 2021-12-17

**Authors:** Alexandria Ebert, Julie Hicks Patrick, Maya Huggins, Aradhita Yadava, Sarah Collett

**Affiliations:** West Virginia University, Morgantown, West Virginia, United States

## Abstract

Increases in motivation for the pursuit of emotionally meaningful goals and activities as people age (socioemotional selectivity theory; SST) necessitates the investigation of means in which these goals and activities can be realized. In the present study adults (N = 130) aged 25 to 78 (M = 53.32; SD = 15.181) watched awe-inducing (view of space) and happiness-inducing (comedian Robin Williams interacting with Koko the “talking gorilla”) videos and then completed measures associated with awe, affect, and well-being (measured via PGC Positive and Negative Affect Scales). Analyses of Variance (ANOVAs) were conducted to examine the effect of age (split by young, middle-aged, and older) on experiences of awe and positive affect in response to watching each video. There was a main effect of age on experiences of awe for both videos (ps < 05). Specifically, older adults experienced significantly higher levels of awe than young adults (p < .05) in response to video 1. They also experienced significantly higher levels of awe than young adults (p < .01) in response to video 2. Bivariate correlations among awe, well-being, and happiness were similar in strength and magnitude in each age group (ps < .05). Overall, consistent with Socioemotional Selectivity Theory, our findings suggest that affective reactions of awe and happiness, induced by videos, relate to well-being across age-groups. Future work should test whether the use of these awe-inducing videos can improve well-being.

